# In canine cranial cruciate ligament disease, are conservative treatment and lateral fabellotibial suture recoveries comparable?

**DOI:** 10.18849/ve.v8i2.612

**Published:** 2023-05-31

**Authors:** Tafara Mapuvire+

**Keywords:** CRANIAL CRUCIATE LIGAMENT DISEASE, SURGICAL TREATMENT, LATERAL FABELLOTIBIAL SUTURE, EXTRACAPSULAR SUTURE, NONSURGICAL TREATMENT, CONSERVATIVE TREATMENT, DOGS

## Abstract

**PICO question:**

In dogs with cranial cruciate ligament disease, is conservative non-surgical treatment as effective as surgical treatment with the lateral fabellotibial suture technique in reducing time to recovery?

**Clinical bottom line:**

**Category of research:**

Treatment.

**Number and type of study designs reviewed:**

One retrospective study.

**Strength of evidence:**

Zero.

**Outcomes reported:**

There is no evidence that surgical treatment with the lateral fabellotibial suture (LFTS) reduces time to recovery compared to conservative treatment in dogs with cranial cruciate ligament disease.

**Conclusion:**

In dogs with cranial cruciate ligament disease there is no statistical evidence to support recommendation of surgical treatment with the LFTS technique compared to conservative treatment in reducing time to recovery.

## How to apply this evidence in practice

The application of evidence into practice should take into account multiple factors, not limited to: individual clinical expertise, patient’s circumstances and owners’ values, country, location or clinic where you work, the individual case in front of you, the availability of therapies and resources.

Knowledge Summaries are a resource to help reinforce or inform decision making. They do not override the responsibility or judgement of the practitioner to do what is best for the animal in their care.

## Clinical scenario

You are presented with a 7-year-old Labrador Retriever with a confirmed complete rupture of the left cranial cruciate ligament. You offer the treatment options available to the client. The client explains that finances are constrained, and you offer either conservative treatment or surgery with the lateral fabellotibial suture (LFTS) technique, both as cost effective options. The client is willing to go with the cost effective surgical option of the LFTS technique if it will result in a quicker recovery than conservative treatment with non-steroidal anti-inflammatories (NSAIDs) and restricted exercise for 6–8 weeks. You look for evidence comparing the rate of recovery of the two treatments in order to be able to advise the client appropriately.

## The evidence

There is no evidence that dogs treated with the LFTS technique to address cranial cruciate ligament disease recover quicker than dogs treated conservatively with NSAIDs and exercise restriction.

## Summary of the evidence

**Chauvet et al. (1996):** 

Population:	Dogs weighing more than 22.7 kg with cranial cruciate ligament ruptures managed with fibular head transposition (FHT), lateral fabellar suture (LFS) or conservative treatment presented at the University of Illinois Veterinary Medical Teaching Hospital between 1986 and 1991.
Sample size:	61 dogs, 72 stifles.
Intervention details:	22 stifles (19 dogs) were treated with fibular head transposition (FHT) technique. Time between intervention and evaluation mean 26.7 months, median 28.5 months, range 3–66 months. 39 (36 dogs) stifles were treated with lateral fabellotibial suture (LFTS) technique. Time between intervention and evaluation mean 20.4 months, median 13.5 months, range 6–60 months. 11 (10 dogs) stifles were treated with conservative treatment (CT). Time between intervention and evaluation mean 7.1 months, median 3 months, range 3–24 months.
Study design:	Retrospective study.
Outcome studied:	Subjective: Owner evaluation outcomes. Investigator physical examination evaluation. Investigator radiographic evaluation. Objective: Investigator force plate evaluation.
Main Findings (relevant to PICO question)	Mean owner evaluation score for conservative treatment was lower than that for LFTS technique but not statistically different. No significant differences in investigator evaluation scores. No statistical difference in force plate peak vertical forces.
Limitations:	Clinical evaluation is subjective. No preoperative force plate evaluation was carried out to provide a reference for comparative purposes. Comparison of force plate evaluation of affected limb with contralateral limb is validated. However, several dogs presented with lower ground reaction forces in the contralateral limb, suggesting possible injury. Ideally comparison should be with an unaffected limb. Although a power analysis was not performed, the sample size could be too small for proper statistical inference. There was no presurgical evaluation of affected limbs to provide a reference for postsurgical evaluation. Sample only included large dogs weighing more than 22.7 kg. It is difficult to conclude whether the results of this study can be applied to a heterogeneous dog population including those weighing less than 22.7 kg. Cranial drawer sign, joint crepitus and capsule thickening are of little value as sole methods for evaluating stifle recovery and may not correlate to functional outcomes. The choice of surgery or conservative treatment was made by mutual agreement between the owners and respective veterinary surgeons. This means that interventions were neither blinded nor randomised. Owner compliance was not evaluated.

## Appraisal, application and reflection

Cranial cruciate ligament (CCL) disease is a leading cause of hindlimb lameness in adult dogs (Ness et al., 1996). Surgical management of CCL rupture (CCLR) is often recommended with a variety of surgical techniques described. Surgical techniques to address CCLR in dogs can be broadly classified into three categories. One category of techniques aims to provide dynamic stabilisation of the CCL deficient stifle by eliminating cranial tibial thrust (CTT). Another category of techniques aims to provide a temporary static fixation by extracapsular stabilisation. The third category of techniques aims to provide a static fixation by replacement of the failed CCL with an intra-articular graft. Evidence based assumptions are difficult to make regarding the preferred surgical treatment option for patients. However most recent reviews appear to support the tibial plateau levelling osteotomy (TPLO) technique (Tikekar et al., 2022; Krotscheck et al., 2016; and Bergh et al., 2014). The TPLO procedure can be considered an advanced procedure with a significant learning curve and the necessity for additional equipment and expense. Extracapsular suture (ECS) techniques such as the lateral fabellotibial suture (LFTS) technique can often be performed with less expensive surgical equipment and implants, and less overall cost to owners. This makes the ECS a viable option for many patients. The goals of surgery to manage CCLR are to provide immediate stifle stability, allow a quick return to optimal function and slow the development and progression of osteoarthritis in the affected joint. A 1984 study by Vasseur demonstrated that small dogs weighing less than 15 kg can do well without surgery. The same study showed that outcomes after conservative treatment were poor for dogs weighing more than 15 kg. Considering this, it remains unclear what the expected outcomes would be in a heterogeneous population of dogs. Clients often opt out of surgery for various reasons including, but not limited to, financial constraints, increased surgical and or anaesthetic risks due to age, and / or presence of other medical, orthopaedic and / or neurologic disease. In these cases, clients might seek to gain more insight into the expected outcomes associated with conservative treatment compared to surgical intervention with ECS in order to decide whether surgical intervention would be worth the accompanying expense and / or risk. The 1996 prospective experimental study by Jevens et al. compared functional outcomes between modified retinacular imbrication technique (MRIT) and conservatively treated dogs with experimentally induced rupture of the CCL. They demonstrated that dogs that underwent MRIT did significantly better than dogs that received conservative treatment according to the results of force plate analysis, although the recovery was prolonged. However, MRIT and LFTS are distinct procedures. The former was first described by Flo in 1975 while the latter appears to be a hybrid of the lateral retinacular imbrication technique first described by De Angelis & Lau in 1970, the MRIT as described by Flo (1975), and the lateral suture technique as first described by Gambardella et al. in 1981. Both the MRIT and LFTS fall under the extracapsular technique classification. The study by Jevens et al. (1996) therefore, may not be relevant to the clinical question. A 1996 retrospective study by Chauvet et al. demonstrated that there was no statistical difference in outcomes for dogs treated for CCL disease, between conservatively and LFTS treated stifles according to investigator evaluation. Although owner evaluation demonstrated that LFTS had higher functional scores than conservative treatment, the difference was not statistically significant. Owner evaluation is subjective and difficult to use as a basis for scientific conclusions. The results of owner evaluation could be attributable to the placebo effect in the surgically treated group as the owners were not blinded as to which treatment their dog had received. The only objective method of outcome analysis in this study was force plate analysis, which showed no statistical difference between the two interventions. The comparison of force plate evaluation of the test limb with the contralateral limb is validated (O’Connor et al., 1989). However, there is no evidence that the contralateral limbs were evaluated before the interventions to ensure that they were normal and therefore, acceptable as bases for comparison. The study also excluded dogs weighing less than 22.7 kg, making it difficult to determine whether the results can be applied to dogs weighing less than 22.7 kg. The recent availability of client based validated metrology instruments such as the Bologna Healing Stifle Injury Index (BHSII [Pinna et al., 2019]), Canine Orthopaedic Index (COI [Brown, 2014a; 2014b; and 2014c]) and the Liverpool Osteoarthritis in Dogs (LOAD [Walton et al., 2013]), can help address the limitation of lack of objectivity in owner evaluation. However, they would be more relevant if used in prospective, randomised and blinded studies. The paucity of such studies renders these instruments less useful. The significance of this when applying the evidence to a heterogenous population of dogs is difficult to determine. Owner compliance in the study by Chauvet et al. (1996) was also not evaluated. This is an important factor affecting outcome and evaluation of compliance should therefore ideally form an integral part of any study aiming to compare outcomes of two interventions. The time from intervention to evaluation also varied widely in the study, making it difficult to determine and compare the extent of reduction of time to recovery of the two interventions under comparison. The PICO question specifically aimed at comparing the reduction in time to recovery. Therefore, time between intervention and evaluation should be standardised in order for the study to be able to address the PICO question. The retrospective nature of the study makes it difficult to address this shortcoming. There is no evidence that points towards shorter recovery times with LFTS. However, evidence can be obtained by using prospective, randomised, blinded and controlled clinical trials with large sample sizes representing a heterogeneous population of dogs with naturally occurring cranial cruciate ligament disease. There is paucity in such studies. The RCVS Knowledge Canine Cruciate Registry aims to plug this evidence base gap by collecting data from practitioners in the UK performing surgery to address cruciate ligament disease in dogs. This initiative may provide the much lacking, albeit needed, evidence to inform treatment choices for practitioners and owners alike (www.caninecruciateregistry.org). Although participation in the initiative is limited to dog owners and practitioners registered and performing cruciate surgery in the UK, results from data collected can, hopefully, be extrapolated to any heterogenous population of dogs worldwide.

## Methodology

**Search strategy:** 

Databases searched and dates covered:	CAB Abstracts on OVID Platform 1973–Week 39 2022 PubMed accessed via the NCBI website 1910– September 2022
Search strategy:	CAB Abstracts: (dog or dogs or canine or canines or bitch or bitches) or exp dogs/ or exp canis/ or exp bitches/ (cranial and cruciate) (lateral or fabellar or fabello-tibial or tibiofabella or extracapsular or extra-capsular or LFTS or de Angelis or 'modified retinacular imbrication' or MRIT ('non operative' or 'non surgical' or conservative or nonoperative or nonsurgical or non-operative or non-surgical or ('anti$inflammatory drug* and analgesi*) or nutraceutical* or 'dietary management' or 'prescription diet*' or 'weight loss' or physiotherapy or 'exercise restriction' or rest) 1 and 2 and (3 or 4) PubMed: dog or dogs or canine or canines or bitch or bitches cranial and cruciate lateral or fabellar or fabello-tibial or tibiofabella or extracapsular or extra-capsular or LFTS or de Angelis or 'modified retinacular imbrication' or MRIT. conservative or nonoperative or nonsurgical or non-operative or non-surgical or (antiinflammatory drug* and analgesi*) or nutraceutical* or 'dietary management' or 'prescription diet*' or 'weight loss' or physiotherapy or 'exercise restriction' or rest 1 and 2 and (3 or 4)
Dates searches performed:	20 Sept 2022

**Exclusion / inclusion criteria:** 

Exclusion:	Irrelevant to the PICO. Articles not in English.
Inclusion:	Articles in English. Articles comparing outcomes of relevant interventions.

**Search outcome:** 

Database	Number of results	Excluded – Article in English	Excluded – Article irrelevant to PICO	Total relevant papers
CAB Abstracts	428	45	382	1
PubMed	280	5	274	1
Total relevant papers when duplicate removed				1

## ORCiD

Tafara Mapuvire: https://orcid.org/0000-0002-7170-0624

## Conflict of interest

The author declares no conflicts of interest.
